# Test Reliability and Compliance to a Twelve-Month Visual Field Telemedicine Study in Glaucoma Patients

**DOI:** 10.3390/jcm11154317

**Published:** 2022-07-25

**Authors:** Selwyn Marc Prea, Algis Jonas Vingrys, George Yu Xiang Kong

**Affiliations:** 1 Department of Optometry and Vision Sciences, The University of Melbourne, Parkville, VIC 3010, Australia; algis@unimelb.edu.au; 2 Glaucoma Investigation and Research Unit, Royal Victorian Eye and Ear Hospital, Melbourne, VIC 3002, Australia; gkong008@hotmail.com; 3 Centre for Eye Research Australia, Royal Victorian Eye and Ear Hospital, Melbourne, VIC 3002, Australia; 4 Ophthalmology, Department of Surgery, The University of Melbourne, Parkville, VIC 3010, Australia

**Keywords:** telemedicine, home monitoring, visual fields, glaucoma progression, guided progression analysis, tablet device, iPad

## Abstract

Background: Our primary aim is to quantify test reliability and compliance of glaucoma patients to a weekly visual field telemedicine (VFTM) schedule. A secondary aim is to determine concordance of the VFTM results to in-clinic outcomes. Methods: Participants with stable glaucoma in one eye were recruited for a 12 month VFTM trial using the Melbourne Rapid Fields (MRF-home, MRFh) iPad application. Participants attended routine 6 month clinical reviews and were tasked with weekly home monitoring with the MRFh over this period. We determined compliance to weekly VFTM (7 + 1 days) and test reliability (false positives (FPs) and fixation loss (FL) <33%). A secondary aim considered concordance to in-clinic measures of visual field (MRF-clinic (MRFc) and the Humphrey Field Analyzer (HFA)) in active participants (≥10 home examinations and 5 reliable HFA examinations). The linear trend in the MRFh mean deviation (MD) was compared to the HFA guided progression analysis (GPA) using Bland–Altman methods. Data are shown as the mean ± standard deviation. Results: Forty-seven participants with a mean age of 64 ± 14.6 years were recruited for the trial. The VFTM uptake was 85% and compliance to weekly home monitoring was 75% in the presence of weekly text reminders in the analysed group (*n* = 20). The analysed group was composed of test subjects with five reliable in-clinic HFA examinations (GPA analysis available) and who submitted a minimum of 10 MRFh examinations from home. Of the 757 home examinations returned, approximately two-thirds were reliable, which was significantly lower than the test reliability of the HFA in-clinic (MRFh: 65% vs. HFA: 85%, *p* < 0.001). The HFA-GPA analysis gave little bias from the MRFh slope (bias: 0.05 dB/yr, *p* > 0.05). Two eyes were found to have clinical progression during the 12 month period, and both were detected by VFTM. Conclusions: VFTM over 12 months returned good compliance (75%) to weekly testing with good concordance to in-clinic assays. VFTM is a viable option for monitoring patients with glaucoma for visual field progression in between clinical visits.

## 1. Introduction

Glaucoma is a progressive optic neuropathy affecting the visual function of 80 million people worldwide [1]. Affected individuals are burdened with frequent clinical visits to monitor their disease progression [2] and to receive tailored medical intervention to reduce the risk of irreversible vision loss. The need for frequent reviews places strain on the healthcare system, resulting in lengthy wait times [3,4]. Furthermore, a proportion of patients with glaucoma experience vision loss due to the fact of delayed follow up [5].

Telemedicine (TM) of visual function is a novel management approach that could potentially reduce some of the shortcomings of standard in-clinic testing with Ganzfeld bowl-based computerised perimetry by providing doctors with supplementary information of a patient’s visual status in-between scheduled clinical reviews. The modelling suggests that clinical progression can be detected earlier with more frequent TM compared to the standard 6 month clinic-based visits [6]. Several options for visual field telemedicine (VFTM) of glaucoma are available using virtual-reality-based headsets [7,8], but these require special purpose equipment for home use. One promising alternative technology is tablet-based perimetry such as the Melbourne Rapid Fields glaucoma iPad application (MRF, Glance Optical Pty., Ltd., Melbourne, Australia) [9,10] and the Eyecatcher [11,12,13]. Tablet technology has the benefit that it can provide a more familiar environment to patients than virtual reality goggles given the ubiquity of smart devices among the population and the fact that many patients may own such devices or be able to borrow them from relatives or friends.

Our previous studies have shown that VFTM with the MRF has excellent uptake and compliance by participants with glaucoma in the short term (6 weeks) [14]. We reported 72% compliance to our request for weekly VFTM and 87% retention to home testing (active testing within 28 days). Despite a large proportion (44%) of home tests having low reliability (FP > 25%; FL > 25%) compared to in-clinic assays with the Humphrey Field Analyzer (HFA, 18%), a strong correlation was found between the mean deviation (MD) of the MRF test and the HFA (r = 0.85) over the 6 weeks of this short-term study [14]. This finding indicates that a large number of tests returned from the weekly VFTMs act to buffer any high levels of intra-test variability [6] to yield reliable outcomes. This is evident in the mean absolute error (MAE) calculation that we reported in that paper as well as by the lower coefficient of repeatability found for the MRF (4.3 dB) compared with the HFA (6.2 dB) [14]. Other research groups have shown excellent test compliance and participant retention to VFTM (98% retention to monthly testing) over a 6 month period [11].

Given these promising preliminary findings we report on a home monitoring trial with telemedicine over the long term (12 months). In this study, we recruited participants who had stable, treated glaucoma at their routine glaucoma review and asked them to perform a weekly VFTM with MRF from home (MRFh) using a loaned iPad tablet and broadband access. In this manuscript, we considered the reliability of tests returned from home and the compliance of participants to the request for weekly testing over a 12 month period. Given the nature of our study population (all had stable glaucoma), in a small number of cases we also consider the capacity of VFTM to; 1. detect early visual field change in comparison to the HFA GPA, and 2. identify progression in comparison to a clinician-based diagnosis derived using standard clinical assays.

## 2. Methods

Ethics approval for this long-term VFTM trial was obtained from the Royal Victorian Eye and Ear Hospital ethics committee (HREC: 12/1220H, approved on 19 May 2020). All experiments were conducted in accordance with the tenets of the Declaration of Helsinki. Informed consent was obtained from all participants prior to enrolment.

### 2.1. Participants

Participants were recruited for this study from the glaucoma clinic of the Royal Victorian Eye and Ear Hospital, Melbourne, Australia, during a routine clinical review. Inclusion criteria were a diagnosis of stable glaucoma in the study eye. The clinical diagnosis was made by a glaucoma specialist who considered results from funduscopy, HFA perimetry, intraocular pressure and optical coherence tomography during routine 6 month clinical reviews, and stability was established by finding no change over the past 2 review periods. The other eye may have been normal; however, if both eyes were diagnosed with stable glaucoma, the eye with the better HFA MD at baseline was selected as the study eye. Additionally, there was a requirement for test subjects to have previously performed at least 2 reliable visual field examinations (24-2 SITA standard and HFA) before entry to our study in order to achieve an adequate number of tests for guided progression analysis (GPA). During the clinical trial, participants undertook a further 3 HFA tests at 6 month intervals to yield the 5 HFA examinations needed for the GPA. This enabled us to generate a slope for the mean deviation (MD, HFA) and establish stability or progression in our participants to compare to MRFh outcomes. During the trial, participants were asked to adhere to their prescribed glaucoma treatment schedule as per the advice from their ophthalmologist. Clinical reviews were conducted at baseline, 6 and 12 months. Exclusion criteria were unstable glaucoma or glaucoma surgery within the past 6 months, a recent change in glaucoma medications, visual acuity worse than 6/12, or the inability to understand English instructions provided by the audio of the iPad application.

### 2.2. Melbourne Rapid Fields App

The Melbourne Rapid Fields software comes in several formats and, here, we report findings for the glaucoma version of this application, which has been described elsewhere [9,10,14]. In short, MRF is an application available for the Apple iPad (Apple, Cupertino, CA, USA) that can be used by participants distant to the clinic in a self-monitoring mode. It has both a polar test grid and a 24-2 test grid and, in this study, we report on the use of the 66 point polar test grid consistent with past publications. Four changes of fixation to each of the corners of the device are required to examine the full extent of visual field. Human visual fields are efficiently thresholded using a three-step Bayesian protocol based on a probability density function determined from approximately 40,000 normal and diseased data points [9].

To facilitate self-monitoring, the MRF has audio prompts in many languages that guide patients through the examination, including requesting fixation changes to test peripheral regions of the visual field as well as in the submission of results. In this trial, we used the English set of prompts and required participants to understand the English language.

The MRF checks for fixation stability when testing during the central fixation phase using a blind spot monitor. This presents stimuli to the blind spot to estimate fixation accuracy. During the peripheral fixation phases, MRF provides audio commands that remind participants to maintain fixation to the red fixation cross. 

Testing conditions at home are standardised by asking the patient to test in a quiet room free from distractions and reflections off the screen. The desired test distance of 33 cm can be achieved by training the patient to place their elbow at the edge of the iPad and using the palm of this hand to cover the non-tested eye.

Test outputs are stored on cloud portal with Health Insurance Portability and Accountability Act (HIPAA) compliant methods, and clinicians can access these results via the online portal, where a decision can be made to review a patient sooner if disease progression is confirmed. For this trial, this early recall option was not implemented: patients were reviewed on a 6 month cycle, and all clinical decisions were based on the data collected at these 6 month visits including HFA outcomes.

### 2.3. Testing Procedures

**Figure 1:**
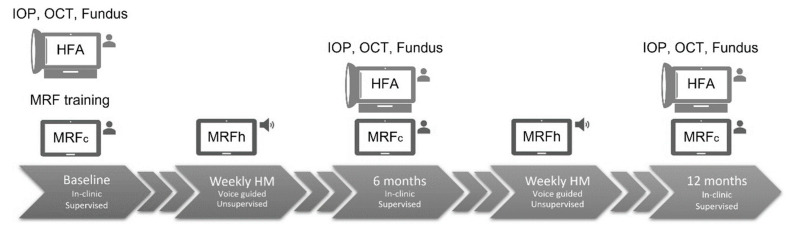
Experiment timeline. In-clinic visits were conducted at baseline, 6 months and 12 months with weekly home monitoring in between. HFA = Humphrey Field Analyzer; MRFc = Melbourne Rapid Fields performed under clinical supervision; MRFh = Melbourne Rapid Fields performed at home under voice guidance; IOP = intraocular pressure; OCT = optical coherence tomography.

Patients were introduced to the MRF as reported elsewhere [14,15] and were given in-clinic supervised training on how to perform the test and to familiarise them to the audio instructions (Figure 1). The training session served as an opportunity for test subjects to receive feedback on their technique for VFTM testing and results submission, and to clarify any outstanding queries with the study coordinator. A loan iPad (iPad Air 2 or iPad Pro, Apple, Cupertino, CA, USA) with cellular broadband connection was allocated to each participant and an appropriate day and time was mutually agreed upon for a text message reminder to be sent for the need for testing. Although participants might have had their own iPad, all appreciated the loaned unit and complimentary broadband connection which we provided as an inducement for continued participation to our trial. A set of step-by-step written instructions along with contact details of the study coordinator were provided for easy resolution of any technical difficulties. 

On the day a home-based examination was due, participants were asked to test in a quiet room free from distractions and light reflections off the tablet screen. A simple technique was demonstrated whereby an elbow could be placed at the edge of the iPad case/keyboard to achieve the correct test distance of 33 cm. The palm of the same hand could be simultaneously used to occlude the non-tested eye. The participant was instructed to wear their habitual near correction for the duration of testing. Test subjects were asked to perform VFTM using MRFh once a week for one year and to ensure that they returned for their 6-monthly clinical reviews (Figure 1). The compliance to clinical review timing was frustrated by the onset of the COVID-19 pandemic as will be detailed later. All loan equipment was returned at the 12 month visit and the study was ended.

### 2.4. Data Analysis

**Figure 2:**
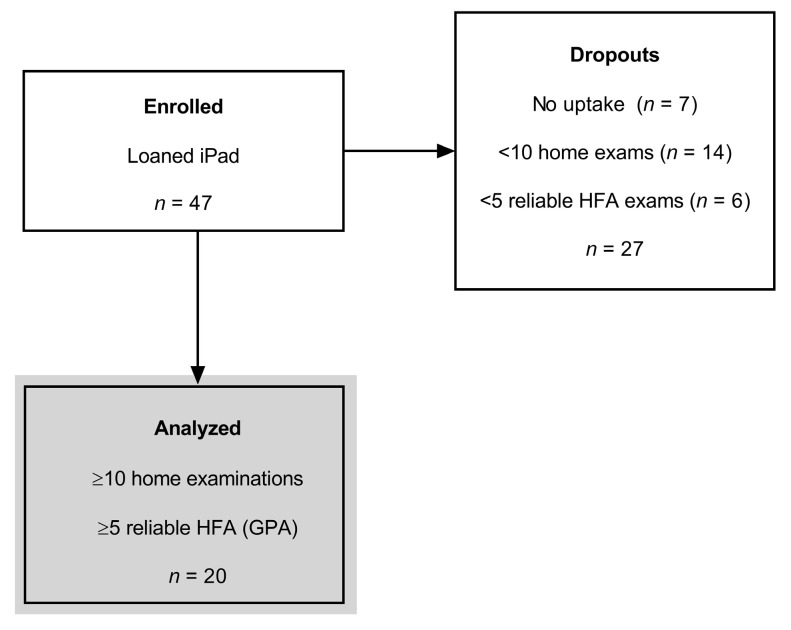
CONSORT diagram for the long-term visual field telemedicine trial. Numbers given are total number of participants. Forty of the forty-seven enrolled participants returned at least one home examination and were included in our analysis of Mean Absolute Error (see Figure 3). Twenty participants performed a minimum of 10 home examinations and provided 5 reliable HFA exams in-clinic (which enabled guided progression trend analysis, GPA). This group was included in the concordance and retention analysis (shaded area). Note, two of the patients in the analysed group showed progression, which will be detailed later.

Data analysis was conducted on the eye with the better HFA MD at baseline. Data collected from participants with uptake of VFTM (≥1 home examination, see Figure 2) were used to calculate the mean average error (MAE, see later). The MAE was calculated from the median MD outcome for each participant, as the median is less affected by outliers. In our analysis, we included data with reliable HFA outcomes (5 tests) and compared to reliable MRFh outcomes returned from home.

Compliance to weekly testing was classified as successful if returning a test within 7 + 1 days of a previous test, as requested on recruitment. MRF test results were considered reliable if the false positive (FP) and fixation loss (FL) rates were below 33% [16]. 

**Figure 3:**
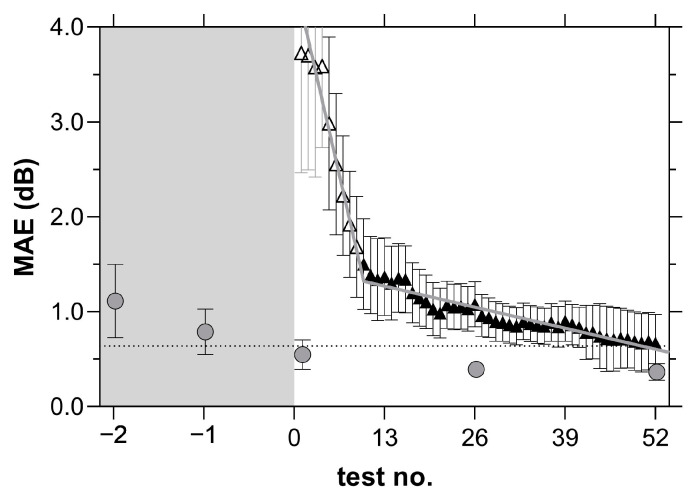
Mean absolute error (MAE, dB) from the median MD value calculated from the average of 5 HFA tests performed in-clinic (filled circles) and the average of 52 MRFh tests performed at home (filled triangles) in *n* = 20 glaucoma participants. The first 9 unfilled triangles represent a period of learning with high deviations from the median value. Error bars represent SEM. The grey curve represents a two-line fit of the MRFh data which assumes a shallower slope after 10 tests. The grey zone on the left indicates the period preceding VFTM where participants were required to have a minimum of 2 reliable HFA examinations to enable guided progression trend analysis.

The MD raw data and time dependent trends generated over the 12 month window of VFTM were calculated individually. MD values were compared to an individual’s median MD over the 12 months by computing the 95th percentile for each MD severity level based on the baseline HFA exam: normal (MD ≥ −2.1 dB, 0.73 dB), mild (−6 dB < MD < −2.1 dB, 3.34 dB), moderate (−12 dB < MD < −6 dB, 4.05 dB) and severe (MD < −12 dB, 3.83 dB). ‘Fluctuation events’ were recorded as greater than the expected variability returned by our test cohorts 95th percentile for their given MD severity level. We classified MD trend as ‘stable’ if there was ≤1 fluctuation event or fluctuating if there was >1 fluctuation event post learning phase (test 10 onwards, see mean absolute error, Figure 3). Fluctuation events could be subdivided into the ‘learning phase’ (tests 1–9, see MAE, Figure 3) and the ‘post learning phase’ (test 10 onwards). 

Outcomes from supervised visual field testing in-clinic (HFA and MRFc) were compared to unsupervised testing from home (MRFh) by performing a t-test on the mean deviation and the pattern standard deviation (or pattern defect for the MRF). Concordance between the HFA and MRFh MD are given by Spearman rank-order correlations (r_s_) after Kolmogorov–Smirnov testing established that the data were non-Gaussian (GraphPad Prism 8.4, San Diego, CA, USA). Test reliability at-home versus in-clinic were compared in a 2 × 2 table with a Fisher’s exact test. The average coefficient of repeatability (CoR) was calculated for each method and significance was tested with an F-ratio.

Concordance of home monitoring with in-clinic HFA outcomes was determined by Bland–Altman analysis comparing the MD slopes for MRFh and the HFA guided progression analysis (GPA, MD slope). The MRFh trend was derived from all data collected over the study window (12 months, 20 participants), whereas GPA data were spread out over a period of 36 months that encompassed the VFTM period. MRFh progression was defined as a linear trend that was ≤−1.25 dB/yr [17], which was calculated post hoc. Two participants were identified as showing progression by a glaucoma specialist who considered results via funduscopy, HFA perimetry, intraocular pressure, and optical coherence tomography during the 6 month clinical reviews and was blinded to the results of the VFTM.

## 3. Results

### 3.1. Uptake, Compliance, and Test Reliability of the VFTM over 12 Months

We enrolled a total of 47 participants (Table 1) in the 12 month VFTM trial, who had a mean age of 64 years (range: 21–89 years, Table 1). The diagnosis in the total group was glaucoma in 70% (POAG + other glaucoma) as shown in Table 1. Four of these participants (seven eyes) also volunteered for our short-term trial (6 weeks) [14] and agreed to continue testing to 12 months. Females represented 26% of the cohort. 

There was uptake in 85% of participants (*n* = 40, Figure 2), whereas *n* = 7 cases failed to return a test outcome from home and were deemed to have dropped out of the study. Six of these seven cases of dropout were not familiar with tablet technology and one case experienced competing life demands which inhibited them from participating. 

Figure 3 describes the MAE analysis of the median MD value for 52 weeks of VFTM. After the 10th home test, a shallower MAE change was observed. A two-line fit to the MAE as a function of test number enabled us to determine the point at which the MAE began to change slope; this was found to be 10 tests for MRFh (Figure 3). Tests 1–9 were therefore defined as a ‘learning phase’, and we excluded *n* = 14 people who did not progress past this phase from further analysis (Figure 2, learning, <10 home examinations).

Furthermore, calculation of the HFA GPA requires a minimum of 5 reliable HFA examinations, and this was not achieved in *n* = 6 individuals (Figure 2, no GPA, <5 reliable HFA examinations). Our analysed group, reported from here onwards, was composed of *n* = 20 participants that progressed passed the ‘learning phase’ and delivered five reliable HFA examinations in-clinic (Figure 2, analysed, grey shaded area).

**Figure 4:**
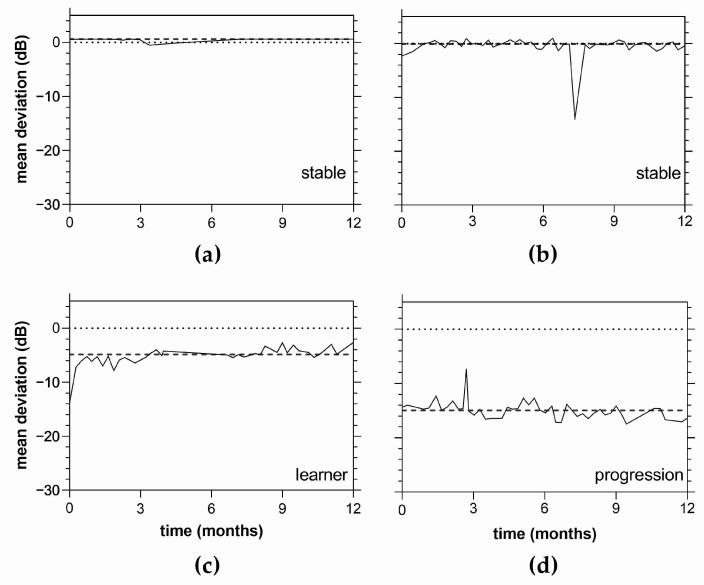
Example mean deviation (MD) trends over 12 months of visual field telemedicine for 4 participants: (**a**) stable data with no learning or fluctuation events; (**b**) stable with a single fluctuation event; (**c**) learning effect with fluctuation; (**d**) one case of progression on the MRFh (Progressor 1, Figure 9). The dashed lines indicate the median of all data points. Although the progression involves only a small change (approximately 2 dB), note how the data consistently falls under the median line towards the end of the monitoring period (right side of (**d**)).

Examples of the raw data (MD trend) for select participants over 12 months of the VFTM are shown in Figure 4. In the post-learning period (test 10 onwards), a significant proportion of participants returned slopes that could be classified as stable and free from fluctuation (55%, ≤1 fluctuation event post learning). The remaining 45% showed fluctuation during this same period (>1 fluctuation event post-learning). A learning effect was observed in 45% of subjects with evidence of substantial fluctuation within the first 9 trials followed by improvement. We believe that the learning effect reflects familiarity with the technology, as all of our cases were experienced with HFA perimetry. This learning effect rendered artefactual improvement in three of the five eyes. Two eyes exhibited progression in their VFTM outcomes, with one example shown in Figure 4d.

**Figure 5:**
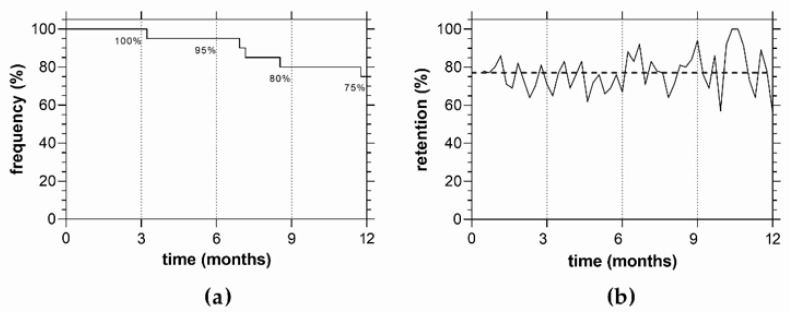
Time to dropout and compliance to weekly testing in *n* = 20 eyes that undertook visual field telemedicine with MRFh: (**a**) time to dropout over 12 months of visual field telemedicine with MRFh; (**b**) compliance to the request for weekly testing (7 + 1 days) returned by active participants over this period. Note the number of active participants decreased to 75% (*n* = 15 of 20) as shown in (**a**).

Of the expected 1040 home examinations (20 active participants × 52 tests), 757 (73%) were returned. By 12 months, a total of *n* = 5 eyes had dropped out leaving 75% of the initial group active at the 12-month time point (*n* = 15, Figure 5a). The average compliance to the request for weekly testing (7 + 1 days, Figure 5b) in the analysed group following text message reminders was high at 75% considered over the entire year. Our analysis of the reasons for lack of compliance found the common causes were IT logistical issues or unscheduled absences (e.g., participant taking a vacation). 

**Figure 6:**
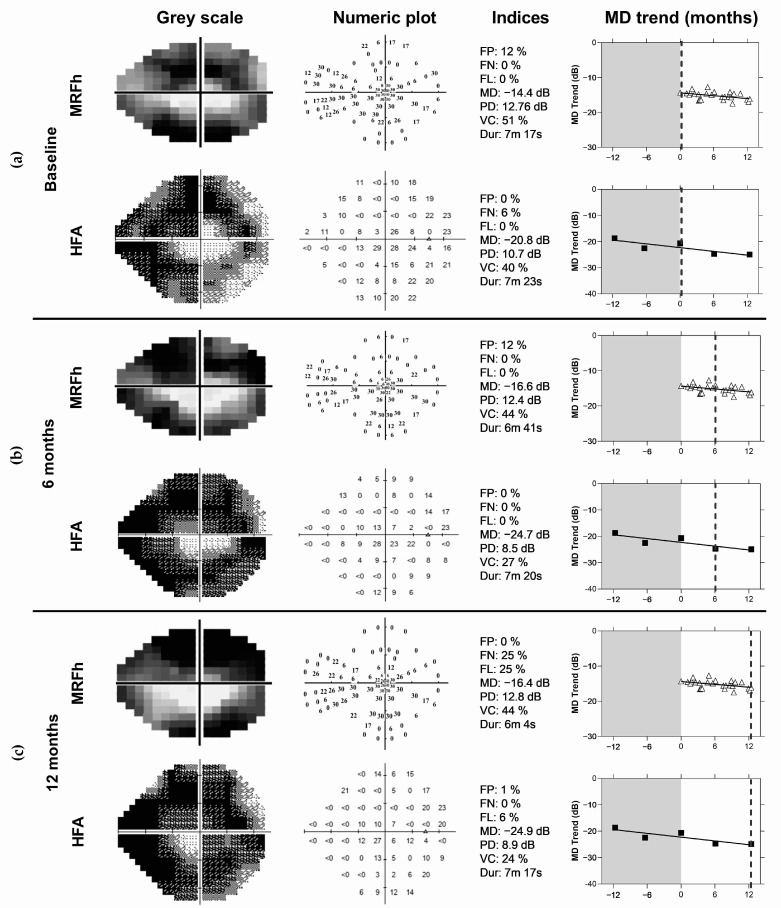
Typical outcome returned after 12 months of the VFTM by a 58 year old with glaucoma progression identified by clinical methods. The MRFh and HFA results are shown at (**a**) baseline, (**b**) 6 months and (**c**) 12 months. Right panels show the mean deviation trend (MD, dB) over 12 months for MRFh (filled triangles) and HFA (filled squares). *x*-Axis labels indicate months. The grey shaded area of the MD trend represents the period prior to VFTM where 2 reliable HFA fields were obtained as a baseline for GPA analysis (5 reliable results in total). The dotted line indicates the timepoint for a representative VFTM result. FP: False positive rate; FN: false negative rate; FL: fixation loss; MD: mean deviation; PD: pattern deviation; VFI: visual field index; VC: visual capacity; Dur: test duration.

Example VFTM results for a 58 year old female progressor are given in Figure 6. A downward trend was observed over the 12 month period of home monitoring (HFA: −2.9 dB/yr, MRF: −1.7 dB/yr). The grey shaded zone in the right panels of Figure 6 indicates the period prior to the VFTM trial, where the participant was required to have shown clinical stability and returned two reliable HFA results for a total of five reliable examinations at the end of the VFTM period enabling GPA analysis (refer to inclusion criteria in Section 2). This was not achieved in 6 of the initial 47 enrolled participants (13%, Figure 2).

### 3.2. Supervised versus Unsupervised Visual Field Testing

**Figure 7:**
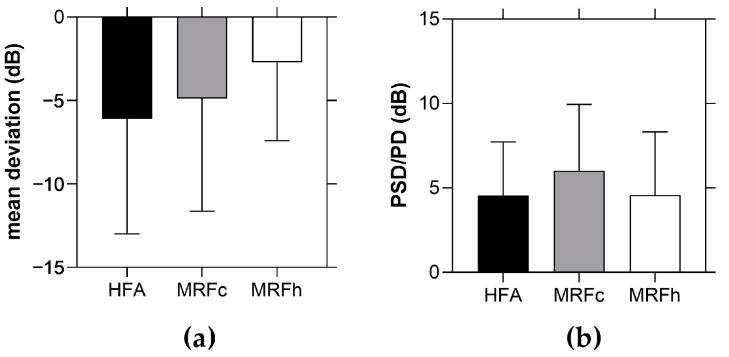
Supervised (HFA, MRFc) versus unsupervised (MRFh) visual field testing: (**a**) average mean deviation for *n* = 20 participants with glaucoma; (**b**) average pattern standard deviation (HFA)/pattern deviation (MRF) from *n* = 20 participants with glaucoma. HFA: Humphrey Field Analyzer; MRFc: MRF performed under supervision by a clinical assistant in-clinic; MRFh: MRF performed under app generated voice prompts from home; PSD: pattern standard deviation (HFA); PD: pattern deviation (MRFc and MRFh).

Given the potential distractions of the home environment, it was necessary to compare unsupervised results undertaken with app generated voice guidance from home (MRFh) with in-clinic testing supervised by a clinical attendant (MRFc and HFA). Figure 7a shows the average MD for each of the three methods, where no significant difference was found in this data (MRFh vs. HFA: *p* = 0.08, MRFh vs. MRFc: *p* = 0.25, MRFc vs. HFA: *p* = 0.59, Figure 7a, Table 2). In addition, the coefficient of repeatability (CoR) for supervised, in-clinic testing (HFA, MRFc) and unsupervised home testing (MRFh) was not statistically different (MRFh vs. HFA: *p* = 0.80, MRFh vs. MRFc: *p* = 0.65, MRFc vs. HFA: *p* = 0.87, Table 2). Similarly, no difference was found between the HFA PSD and the MRF PD (MRFh vs. HFA: *p* = 0.98, MRFh vs. MRFc: *p* = 0.25, MRFc vs. HFA: *p* = 0.21, Figure 7b, Table 2).

Our criteria for a reliable visual field examination (MRFh and HFA) were FL and FP ≤33% [16]. With these criteria applied to both device outcomes, we found that 65% of all home examinations were reliable, which was lower than that found with the HFA performed with clinical supervision (85%, Fisher’s exact test, *p* = 0.38, Table 3). Despite the larger numbers of unreliable tests returned from home, there was a substantial correlation between HFA and MRFh MDs at test 1 (r_s_ = 0.81, (95% CI: 0.55 to 0.93)) and test 10 (r_s_ =0.90, (95% CI: 0.75 to 0.96)).

**Figure 8:**
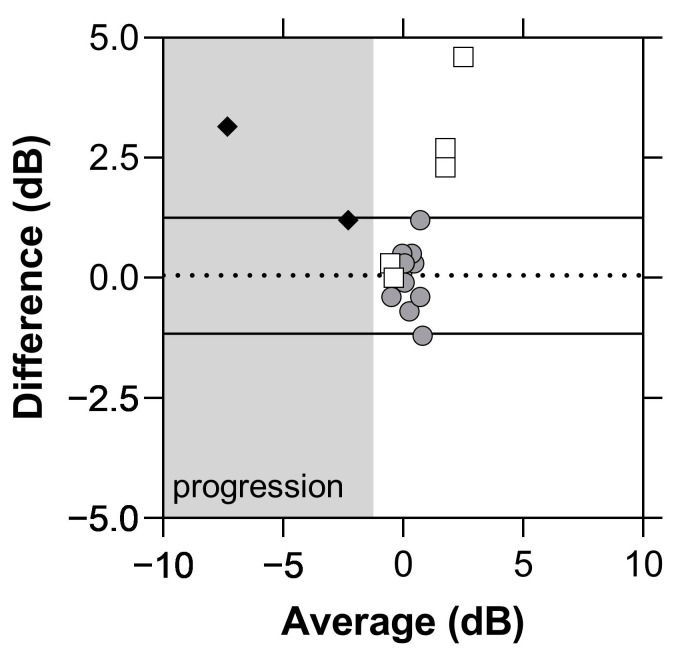
Bland–Altman plot of the HFA guided progression analysis (GPA, MD slope, dB/yr) and MRFh MD trend in glaucoma participants with no progression (filled circles, *n* = 18). Progressors (grey shaded area, black diamonds, *n* = 2) were not included in the Bland–Altman analysis. The unfilled square symbols identify 5 cases who showed significant learning effects (change or fluctuation > 3.7 dB over the 12 month period). Some of these cases returned ‘improved’ thresholds at later times, which produce an artefactual improvement in their outcomes. Average difference: 0.05 dB/yr. 95% Limits of agreement: −1.1 to 1.2 dB/yr.

To establish concordance of the VFTM to in-clinic measures, we calculated the MRFh MD trend based on the total number of home examinations received over the study period. The difference in the slopes returned by the two methods was not significant (0.05, *p* = 0.05) with limits of agreement (LoA) being −1.1 to −1.2 dB/yr (Figure 8). 

### 3.3. Detecting Change with Visual Field Telemedicine

**Figure 9:**
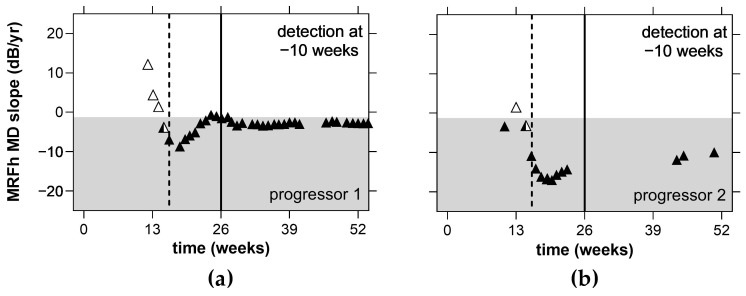
Detecting change in two cases with progression identified by the reviewing clinician. The MRFh MD slope was calculated over a minimum of ≥5 reliable tests. Unfilled triangles represent non-progressing slopes (>−1.25 dB/yr) and semi-filled triangles represent unconfirmed change (single point ≤−1.25 dB/yr). Filled triangles are slopes that indicate confirmed change (second consecutive result ≤−1.25 dB/yr, grey shaded area). Dashed lines indicate confirmed progression at retest. Solid lines indicate next clinical review. Average MD trends for (**a**) progressor 1: MRFh = −2.4 dB/yr, HFA GPA = −2.2 dB/yr; (**b**) progressor 2: MRFh = −10.2 dB/yr, HFA GPA = −1.3 dB/yr).

At this juncture it needs to be recalled that our test cohort were at low risk for progression, as they were all stable, treated glaucoma patients. Nevertheless, over the 12 month VFTM trial, two participants exhibited progression as identified by a glaucoma specialist during one of the regular clinical reviews. VFTM with the MRFh also confirmed progression in these same eyes after 16 weeks (4 months; black, dotted line; Figure 9a,b. This was 10 weeks earlier than the next scheduled clinical review at the 6 month timepoint (solid line; Figure 9a,b) where the clinician identified change. In contrast, the HFA GPA would take a minimum of 2 years (104 weeks, 5 reliable results at 6 month intervals) to detect progression.

## 4. Discussion

The VFTM with a tablet device is a potentially useful method for identifying progression in glaucoma in-between scheduled hospital/clinical reviews. Testing can be completed at a time that is convenient for the patient using equipment that is relatively inexpensive and readily available. In this study, we report on the compliance and reliability of home test results from a cohort of glaucoma patients and the concordance of these results to in-clinic observations made by a glaucoma specialist during routine 6-monthly reviews and outcomes returned by HFA GPA from testing performed at these reviews. 

At the conclusion of 12 months of VFTM, we found a compliance rate to weekly testing of 75% in our analysed group, similar to what we reported in our short-term study (72%) [14]. It should be noted, however, that only 32% of enrolled subjects (15/47) made it to the end of the trial. As detailed in a previous publication, the lack of compliance arises due to the fact of time constraints, IT logistical reasons or lack of motivation [14], and the common rate of compliance between the 6 week and 12 month studies suggests that these factors manifest early during the home monitoring cycle. Moreover, we found that 25% of our cases showed a learning effect that we believe reflects an inability to cope with the technology and that this effect created artifactual improvements in their outcomes. It is our opinion that training and close monitoring of these people might result in better outcomes earlier. Given this finding, close supervision early during home monitoring and training would help improve test reliability.

Although our data found modest compliance to weekly testing, another study that considered tablet-based home monitoring of visual field in glaucoma patients found an adherence rate of 98% to monthly testing [11]. Post hoc analysis of our data found a 97% adherence rate when analysed on a monthly cycle consistent with this past analysis. With a weekly compliance rate of 75%, on average, we can expect to receive approximately 40 reliable examinations from our patients each year when weekly testing is requested. The MAE analysis suggests that a slow progressor (−0.8 dB/yr) [17] can be detected after 10 home tests given that the MAE reduces. This would help doctors identify progression early (as evident by the dotted lines in Figure 9a,b), prompting an unscheduled clinical review, and resulting in better visual outcomes for patients. Conversely, patients with stable fields and no progression may have their clinical reviews extended beyond 6 months, freeing up valuable hospital and clinical resources. It should be noted that variability was reduced after 10 VFTM examinations. This suggests that prior to 10 examinations, only large changes in MD can be detected, whereas after 10 examinations, smaller changes in MD can be exposed. We chose to analyse concordance by correlating the MD trend of the MRFh to the MD trend generated by the HFA GPA. A moderate correlation is observed (R = 0.05, 95% LoA: −1.1 to 1.2 dB/yr, Figure 8), which is likely due to the differences in spot size, spot location and background luminance of the MRFh. Elsewhere, we showed that larger spots produce higher thresholds and reduce threshold variability [19]. Furthermore, other research groups have reported that larger size V spots performed as well as size III spots for longitudinal glaucoma progression analysis likely due to the reduced variability [20]. Our present data indicate a similar level of variability (Table 2, CoR) which might arise from the smaller sample size of our current study as we have shown that this is the case in past clinical trials based on larger samples (see ref. [14]).

At the end of this VFTM trial, disease progression as defined at routine clinical review by a glaucoma specialist was identified in two eyes of two participants. The diagnoses were made using standard clinical methods which included dilated fundus examination, optical coherence tomography (OCT) and VF examination (HFA, GPA). Home monitoring with the MRFh was able to identify change in both patients after 16 weeks (Figure 9a,b) which was 10 weeks prior to the next clinical visit. In contrast, the HFA GPA requires 104 weeks to detect change in these same patients assuming 6 month reviews (64 weeks with 3 month reviews). In addition, reliable VF test outcomes at each review and that a stable baseline was established in the first two tests. It should be noted that only 50% of our cohort (20/40 participants with VFTM uptake) returned five reliable HFA tests over the study period enabling GPA analysis, compared to 65% of MRFh tests being available.

Rather than the intensive 12 months of weekly home monitoring that we set out to achieve, our data show that short, intense periods of testing (14–20 weeks) will expose true progressors and retain high levels of interaction (≈75%, Figure 5b). An alternative monitoring approach would be to adopt a shorter testing interval (<1 week) over a 1 to 2 week period to overcome the initial ‘learning phase’ and reduce the MAE, and then revert to monthly testing thereafter. In the presence of suspected change flag, a shorter testing interval could be adopted again (perhaps the interval may be shortened based on how much the results have deviated from baseline) to either confirm or reject the prospect for change. Both approaches would provide high levels of interaction with the best chance of detecting a progressor. This alternate approach is similar to the ‘wait and see’ concept proposed by Crabb and Garway-Heath [21].

A limitation of this study was that it was undertaken during the pandemic, which would make it more likely for higher compliance rates to be achieved. Whilst a benefit of the study is that participants were able to continue testing and receiving feedback on their ocular condition during times where hospital reviews were limited, the fear of attending a hospital setting where contracting the virus was high coupled with the free access to the device may have boosted compliance levels. Therefore, our weekly compliance finding of 75% may in fact be lower outside of a pandemic. Despite this, our compliance rates are similar to those reported by other studies [14,22], and Figure 5 shows that our 6 month compliance rate (95%) was similar to that found outside of a pandemic [11]. Another limitation of this study was the temporary closure of our research department due to the pandemic. This prevented us from recruiting more participants, therefore, limiting our ability to investigate change detection.

One important observation from our study is that even though our research office was closed with limited hospital appointments during the pandemic, participants already enrolled in the study were able to continue home monitor of their vision and submit test results to the study coordinator during periods of lockdown brought on by the pandemic. This demonstrates the value of VFTM as possible with the MRF and when applied to a chronic disease such as glaucoma. Of note for tele-ophthalmology, MRF software is now available as an online browser option (not evaluated in this study) allowing patients to do the testing at home on their own personal computer [23].

To ensure success of VFTM, we advocate the need for a ‘coordinator’ to actively interact and oversee patient testing at home and to provide the learning and support needed by patients. This could be the same person who would otherwise undertake the visual field testing in the clinic. In this trial, the study coordinator was present at recruitment and performed the MRF training session, where a rapport was developed with the participant. The study coordinator also sent out personalised text messages to each participant the day a test was due and served as a port of call between clinical reviews. Patient feedback was that they liked this personalised attention. One positive outcome of our trial was that numerous participants reported they felt more confident in performing HFA examinations due to the familiarity that they gained on testing with the MRF at home. 

## 5. Conclusions

VFTM is a viable option for monitoring progression in between clinical visits in patients with glaucoma. In the long term (12 months), we found good compliance to weekly testing (75%) in the presence of text message reminders, though test reliability was moderate (at 65%) when testing at home under the guidance of audio prompts. It should be noted that only 50% of the initial group overcame the learning phase and gave five reliable HFA examinations to enable comparison of MRFh to HFA-GPA. The variability (MAE) of the MD decreased after 10 tests suggesting that clinicians may wish to adopt a daily test protocol for the first 1–2 weeks whilst compliance and motivation is high and switch to a monthly test protocol thereafter. 

A strong level of agreement was observed between the MD trend of the MRFh, and the MD trend generated by the HFA GPA. During this study, two participants progressed and VFTM was able to detect changes in both cases before the next scheduled clinical review. VFTM may be a valuable tool in monitoring progression in glaucoma in between scheduled clinical visits. Larger clinical trials are required to fully understand its capabilities and limitations for detecting progression.

## Figures and Tables

**Table 1:** Patient demographics for compliance study.

Demographics		*n* (Total Group, %)	*n* (Analysed Group, %)
Test subjects		47	20
Age, y (minimum–maximum)		64 (21–89)	64 (29–89)
Sex (female)		12 (26)	7 (35)
Diagnosis			
POAG		18 (38)	10 (50)
Other glaucoma ^1^		15 (32)	4 (20)
GS		12 (26)	6 (30)
Normal		2 (4)	--
IOP (mmHg)			
>21		2 (4)	0 (0)
16–21		16 (34)	6 (30)
10–15		27 (57)	12 (60)
<10		2 (4)	2 (10)
VF Severity ^2^	Criteria (dB)		
Normal range	(MD ≥ −2.1) ^3^	23 (49)	8 (40)
Mild	(−6 < MD < −2.1)	8 (17)	3 (15)
Moderate	(−12 < MD < −6)	9 (19)	5 (25)
Severe	(MD < −12)	7 (15)	4 (20)

POAG = primary open-angle glaucoma; GS = glaucoma suspect; IOP = intraocular pressure on the first HFA examination. Visual acuity was better than or equal to 6/12 in the study eye for all participants. ^1^ Other glaucoma includes uveitic glaucoma, normal tension glaucoma, traumatic glaucoma, pseudoexfoliative glaucoma, pigment dispersion glaucoma, neovascular glaucoma and anterior segment dysgenesis. ^2^ Severity based on mean deviation of first HFA examination. ^3^ The definition of the normal range was adopted from Saunders et al [18].

**Table 2:** Mean deviation, pattern standard deviation and coefficient of repeatability of supervised visual field testing in-clinic (HFA, MRFc) and unsupervised visual field telemedicine (MRFh).

	Avg PSD/PD (SD), dB	Avg MD (SD), dB	CoR MD, dB
HFA	4.6 (3.2)	−6.1 (2.7)	7.6
MRFc	6.0 (3.9)	−4.6 (3.6)	10.1
MRFh	4.6 (3.7)	−2.7 (3.3)	9.2

HFA: Humphrey Field Analyzer; MRFc: MRF performed under supervision in-clinic; MRFh: MRF performed under voice guidance at home; CoR: Coefficient of repeatability.

**Table 3:** Telemedicine test of reliability for *n* = 20 glaucoma participants.

	MRFh (% Reliable)	HFA (% Reliable)
FL	78	86
FP	86	98
Total reliable results (FL + FP)	65	85

FL: fixation loss; FPs: false positives; criteria for reliability = FL and FP < 33%.

## Data Availability

A publicly archived dataset generated during the study is available at the following https://doi.org/10.6084/m9.figshare.19100399 (accessed on 10 June 2022).
